# Processing of Biltong (Dried Beef) to Achieve USDA-FSIS 5-log Reduction of *Salmonella* without a Heat Lethality Step

**DOI:** 10.3390/microorganisms8050791

**Published:** 2020-05-25

**Authors:** Caitlin E. Karolenko, Arjun Bhusal, Jacob L. Nelson, Peter M. Muriana

**Affiliations:** 1 Robert M. Kerr Food & Agricultural Products Center, Oklahoma State University, Stillwater, OK 74078, USA; caitlin.e.karolenko@okstate.edu (C.E.K.); arjun.bhusal@okstate.edu (A.B.); jacob.nelson@okstate.edu (J.L.N.); 2 Department of Animal and Food Sciences, Oklahoma State University, Stillwater, OK 74078, USA

**Keywords:** *Salmonella sp.*, 5-log reduction, biltong, dried beef, antimicrobial, water activity, relative humidity, acid adaptation

## Abstract

In the US, dried beef products (beef jerky) are a popular snack product in which the manufacture often requires the use of a heat lethality step to provide adequate reduction of pathogens of concern (i.e., 5-log reduction of *Salmonella* as recommended by the United States Department of Agriculture Food Safety and Inspection Service (USDA-FSIS)). Biltong, a South African-style dried beef product, is manufactured with low heat and humidity. Our objectives were to examine processes for the manufacture of biltong that achieves a 5-log reduction of *Salmonella* without a heat lethality step and with, or without, the use of additional antimicrobials. Beef pieces (1.9 cm × 5.1 cm × 7.6 cm) were inoculated with a 5-serovar mixture of *Salmonella* (*Salmonella* Thompson 120, *Salmonella* Heidelberg F5038BG1, *Salmonella* Hadar MF60404, *Salmonella* Enteritidis H3527, and *Salmonella* Typhimurium H3380), dipped in antimicrobial solutions (lactic acid, acidified calcium sulfate, sodium acid sulfate) or water (no additional antimicrobial), and marinaded while vacuum tumbling and/or while held overnight at 5 °C. After marination, beef pieces were hung in an oven set at 22.2 °C (72 °F), 23.9 °C (75 °F), or 25 °C (77 °F) depending on the process, and maintained at 55% relative humidity. Beef samples were enumerated for *Salmonella* after inoculation, after dip treatment, after marination, and after 2, 4, 6, and 8 days of drying. Water activity was generally <0.85 by the end of 6–8 days of drying and weight loss was as high as 60%. Trials also examined salt concentration (1.7%, 2.2%, 2.7%) and marinade vinegar composition (2%, 3%, 4%) in the raw formulation. Nearly all approaches achieved 5-log_10_ reduction of *Salmonella* and was attributed to the manner of microbial enumeration eliminating the effects of microbial concentration on dried beef due to moisture loss. All trials were run as multiple replications and statistical analysis of treatments were determined by repeated measures analysis of variance (RM-ANOVA) to determine significant differences (*p* < 0.05). We believe this is the first published report of a biltong process achieving >5.0 log_10_ reduction of *Salmonella* which is a process validation requirement of USDA-FSIS for the sale of dried beef in the USA.

## 1. Introduction

Dried meats are a popular food throughout the world due to shelf stability and nutrient content of the food product [1]. Drying and curing of meats dates back centuries as an effective way to preserve foods, particularly meat [2]. The drying of meats allows for reduction of moisture, creating a product with a low water activity (A_w_) and therefore a microbially safe and shelf-stable product as less water is available for microbial growth [1]. An example of a dried meat product is biltong, a ready-to-eat dried beef product native to South Africa. Biltong is usually made from lean strips of beef marinated in traditional spices (coriander, black pepper, salt) and vinegar (malt vinegar, red wine vinegar) and then dried at ambient temperature and humidity following marination [3]. The microbial safety of biltong is due to the combination of vinegar (contributes to low pH), salt (binds water), and drying at low humidity (leads to low A_w_) that inhibits the growth of microorganisms [4,5,6,7]. This style of dried beef is normally produced in a home setting. In the USA there has been a recent surge of interest from both entrepreneurs and commercial manufacturers to produce biltong using traditional methods.

Traditional South African biltong differs from American-style beef jerky mainly due to the absence of a heat lethality step. Beef jerky produced in the US generally includes a heat step to achieve the recommended 5-log reduction of the most likely foodborne pathogen, *Salmonella* [8,9,10]. The lack of a heat lethality step and incubation at ambient temperature during the processing of biltong raises concern for the safety of biltong in regards to potential pathogenic survival and microbial growth such as *Salmonella* spp. and *Listeria monocytogenes* [7]. Since biltong processing does not include a heat lethality step, but rather uses low temperature and humidity conditions to dry meat over an extended period, other ingredients must be used to achieve a microbially safe product. Biltong production utilizes salt, spices, and more importantly vinegar as one of the main additives to impart flavor and reduce microbial survival. Beef jerky in contrast, does not traditionally use vinegar but rather, uses heating, curing, smoking and drying techniques to achieve a microbially safe product [11]. Ultimately, both processes must demonstrate their ability to produce a product that is safe for consumers.

In the US, beef jerky is manufactured under United States Department of Agriculture Food Safety and Inspection Service (USDA-FSIS) compliance guidelines which state that relative humidity during the production of the beef product must be maintained at 90% or above and this humidity can be achieved by use of a sealed oven or continuously injected steam [10]. If these cooking/heating guidelines are not met, a microbial validation of the process must be provided to demonstrate sufficient reduction of the targeted foodborne pathogen. *Salmonella* has been historically associated with outbreaks related to beef [12] or dried beef products [13,14]. Although biltong processing does not maintain high humidity and heat, the manufacturing process must still demonstrate *Salmonella* lethality. Two options are available to achieve USDA-FSIS process validation for biltong. One option is to demonstrate a 2-log reduction of *Salmonella* and perform testing of every lot of edible ingredient to ensure the absence of *Salmonella* as was developed by the ‘Blue Ribbon Task Force’ circa 1996 for *E. coli* O157:H7 [15]. For industry, this option is very difficult because if someone ‘forgets’ to carry out *Salmonella* testing on a given lot of product, or if the test result is positive, it creates a burden for the company, as well as the cost of all the *Salmonella* testing. The second option was that the process itself must provide a 5-log reduction of *Salmonella*, and even though this may be a tough target to achieve, once defined, it is the simplest to implement. The emphasis on achieving a 5-log reduction of *Salmonella* is paramount for the manufacture of biltong that is microbially safe. There are no published reports validating the biltong process in regards to pathogen reduction according to USDA-FSIS guidelines. USA-based studies have not achieved the required 5-log reduction of *Salmonella* [16] and manufacturers in other countries are not required to obtain a specified process reduction by their regulatory agencies [5,6]. Note: USDA-FSIS does not consider the 5-log reduction a ‘requirement’ because of the alternative 2-log process that could be used along with *Salmonella* testing of ingredients; however, if one were choosing the approach without *Salmonella* testing, then by default one would be required to demonstrate a 5-log process.

The purpose of this study was to examine various processes, antimicrobials, and conditions used for the manufacture of biltong to achieve a 5-log reduction of *Salmonella* without a heat lethality step to produce a microbially safe product that could satisfy USDA-FSIS process validation requirements.

## 2. Materials and Methods

### 2.1. Bacterial Strains and Growth Conditions

Active cultures were grown in tryptic soy broth (TSB, BD Bacto BD211825, Franklin Lakes, NJ, USA) in 9 mL tubes at 37 °C. Cultures were maintained for storage by centrifugation (6000× *g*, 5 °C) of 9 mL of fresh, overnight cultures and cell pellets were resuspended in 2–3 mL of fresh sterile TSB containing 10% glycerol. Cell suspensions were placed into glass vials and stored in an ultra-low freezer (−80 °C). Frozen stocks were revived by transferring 100 µL of the thawed cell suspension into 9 mL of TSB, incubating overnight at 37 °C, and sub-cultured twice before use. Microbial enumeration was carried out on tryptic soy agar (TSA, BD Bacto; 1.5% agar) and plated in duplicate.

*Salmonella* serovars used in this study included: *Salmonella enterica* subsp. *enterica* serotype Thompson 120 (chicken isolate), *Salmonella enterica* subsp. *enterica* serotype Heidelberg F5038BG1 (ham isolate), *Salmonella enterica* subsp. *enterica* serotype Hadar MF60404 (turkey isolate), *Salmonella enterica* subsp. *enterica* serotype Enteritidis H3527 (phage type 13a, clinical isolate) and *Salmonella enterica* subsp. *enterica* serotype Typhimurium H3380 (DT 104 clinical isolate). These are well-characterized strains that have been used in numerous research publications involving antimicrobial interventions against *Salmonella* spp. [17,18,19,20,21].

Acid adaptation’ of *Salmonella* serovars was reported in the 1990s by Foster [22] and Leyer and Johnson [23] whereby *Salmonella* pre-exposed to low pH for several doublings during growth were more resistant to low pH than non-adapted cells. Acid adaptation of our *Salmonella* serovars was carried out according to Wilde et al. [24] as modified by Karolenko et al. [21] in which these cultures were inoculated in TSB augmented with 1% glucose prior to use in various conditions of stress.

In preparation of acid-adapted cultures for use in biltong beef processing, individual cultures were propagated overnight at 37 °C in 9 mL TSB (BD Bacto BD286220). These cultures were then used to re-inoculate individual 250 mL centrifuge bottles containing 200 mL pre-warmed TSB containing 1% glucose (BD Bacto BD286220 + 1% glucose) which were again incubated overnight at 37 °C for approximately 18 h. Individual cultures were harvested by centrifugation, and resuspended with 0.1% buffered peptone water (BPW, BD Difco), mixed in equal proportions, and held refrigerated (5 °C) until use shortly thereafter. USDA-FSIS ‘highly recommends’ the use of acid-adapted cultures when such inoculum strains would be used for stressed conditions to insure that they are not easily overcome by acidic processing conditions [25].

### 2.2. Beef Handling and Inoculation

**Figure 1:**
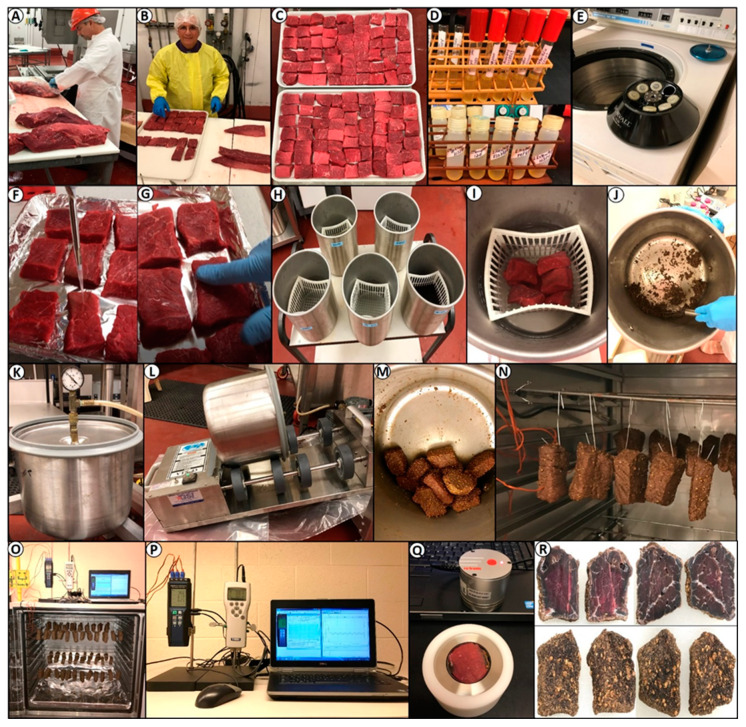
The biltong process: (**A**–**C**) Trimming beef bottom rounds into beef slices and final trimming into squares; (**D**,**E**) acid adapting cultures by growth in tryptic soy broth (TSB) with 1% glucose and centrifuging to concentrate them; (**F**,**G**) pipette inoculation of beef and ‘gloved finger’ spreading of inoculum; (**H**,**I**) baskets and containers for water/antimicrobial dip treatment of inoculated beef; (**J**) mixing vinegar and spices; (**K**–**M**) vacuum tumbling spices and beef; (**N**) hanging beef in humidity chamber; (**O**,**P**) humidity chamber with 4-channel temperature recorder and humidity probe); (**Q**) water activity meter; (**R**) biltong internal and external surface after 8 days of drying.

Beef was processed in the Robert M. Kerr Food and Agricultural Products Center (FAPC; Oklahoma State University, Stillwater, OK, USA). Boneless beef round (i.e., outside round, flat), as per USDA Institutional Meat Purchase Specifications 171B [26] of either Select grade or ungraded were used. Beef was obtained for biltong trials from a local processor (Ralph’s, Perkins, OK, USA) who obtained their beef through a broker that acquires beef from different source companies (i.e., Excel/Cargill, Dodge City, KS, USA; Kane Beef, Houston, TX, USA; High River Angus, Lake River, FL, USA; and others). Boxed, vacuum-packaged beef was held in our meat pilot plant coolers for 1–3 days when received (35 °F/1.7 °C), then trimmed to remove excess fat and sliced by our in-house meat-processing specialists in the FAPC meat pilot plant (Figure 1A).

Refrigerated, trimmed beef was subjected to a ‘final trimming’ prior to biltong processing to further approximate similar-sized pieces of ~1.9 cm thick × ~5.1 cm wide × ~7.6 cm long (~80–110 g) (Figure 1B,C). Inoculum cultures were grown as described earlier, acid adapted, centrifuged, and resuspended to a higher concentration (Figure 1D,E). Individual beef pieces on trays were inoculated with 150 µL (>2.0 × 10^9^ cfu/mL) of the mixed-serovar *Salmonella* cocktail on each side. A ‘gloved finger’ was used to spread the inoculum over the side of the beef pieces, which were turned over and the same inoculation process was performed on the other side (**F**–G). Inoculated beef pieces were then laid flat on foil-lined trays in a refrigerator (5 °C) to promote attachment for 30–60 min prior to use.

### 2.3. Biltong Processing: Antimicrobials

Beef processors are allowed to incorporate an antimicrobial treatment into their process to enhance *Salmonella* reduction. Some meat processors apply this treatment early on with intact beef bottom rounds where *Salmonella*, if present, would likely be on the surface of the beef. Treatment of intact bottom rounds prior to trimming would address *Salmonella* prior to further distribution onto beef surfaces by cutting/trimming, or onto equipment surfaces by additional contact contamination. Still, other processors trim intact bottom rounds into long strips first, and then proceed to take the strips into antimicrobials directly before continuing with marination, tumbling, and drying; this ensures every bit of resulting beef to have been dipped into antimicrobial. For the purposes of experimental methodology, we follow the latter method as it would be impossible to inoculate intact bottom rounds, remove fat, and trim it to size, and follow the originally inoculated surfaces throughout the process. USDA-FSIS has indicated (personal communication) that the later method also mimics the inoculated surfaces of the former method, so our method accommodates both types of commercial applications. USDA-FSIS publishes a list of ‘Safe and Suitable Ingredients for use on Meat and Poultry Products’ that lists various allowable antimicrobials, use level, and product contact time to be considered as processing aids for various types of meat/poultry products [27]. The antimicrobials that were used in this study were chosen from this list (Table 1).

After inoculation and refrigerated hold time to promote bacterial attachment, inoculated beef pieces were dip treated (30 or 60 s) in various antimicrobial solutions (Table 1) using 15.25 × 15.25 × 15.25 cm perforated baskets and cylindrical stainless steel containers (Figure 1H,I); water dip treatment was used as a non-lethal dip control solution. An electronic timer was used to time the process while manually rotating the basket to ensure beef pieces would not be pinned up against each other. After the prescribed time, baskets were removed from the solutions and positioned above the container to allow excess liquid to drip into the container (~20–30 s); samples were then removed individually by sterile forceps into the stainless steel marinade tumbler container for either vacuum- or non-vacuum tumbling (Figure 1J–M).

Antimicrobial (working stock) solutions included 5% lactic acid (FCC88, ADM Co., Decatur, IL, USA), 3% sodium acid sulfate (pHase, Jones-Hamilton Co., Walbridge, OH, USA), acidified calcium sulfate (diluted 2:1 with water: Mionix RTE-17 and Mionix RTE-01; Mionix LLC, Grain Valley, MO, USA) (Table 1).

### 2.4. Biltong Processing: Marination and Drying

Biltong beef marination, depending on the process, generally consisted of addition of a predicated amount of spices (coriander as the predominant spice, black pepper, and others), salt (generally ~2% total formulation), and vinegar (generally ~2% total formulation). An examination of 7 biltong recipes on the internet showed a variety of ingredient formulations that included: beef (82%–96% of total formulation), vinegar (2%–11%), salt (1.5%–8%), coriander (0.3%–2%), pepper (0.1%–1%), brown sugar (0.8%–6%), and bicarbonate (0.2%–1%); our use levels in this study fell within these common values. In addition to adding spices individually, some companies provide premixed biltong ingredients (i.e., Crown National, Freddy Hirsch, Tongmaster). Numerous biltong processes available on the internet also range from short marination periods (30 min to 4 h) to overnight marination (12–24 h); we used representative processes of both of these in our study.

#### 2.4.1. Short-Term Biltong Marination Process

A short marination process was examined for biltong processing consisting of dipping inoculated beef into an antimicrobial solution (or water) for 30 or 60 s, and removing the basket and allowing excess liquid to drip for an additional 30–60 s. After dip treatment, beef pieces were transferred to a stainless steel vacuum chamber containing a biltong spice blend of 1.7%–2.7% salt and 2–4% of 50- or 100-grain red wine (or white) vinegar (as a % of total weight of ingredients including beef). The chamber was evacuated to 38.1 cm (15 inches) Hg, and tumble-marinaded for 30 min on a rotating Biro VTS-43 tumbler (Biro, Marblehead, OH, USA) before hanging to dry. Beef pieces were hung in a temperature-controlled humidity oven (Hotpack, Warminster, PA, USA) at 25 °C (77 °F) and 55% relative humidity (RH) and allowed to dry for up to 8 days. Beef was sampled after inoculation (0 days), after antimicrobial (or water) dip treatment, after marination, and after 2, 4, 6, and 8 days of drying (or until >5-log reduction of *Salmonella* was obtained). The impact of both vinegar and salt was also examined via same-day processes comparing 2%, 3%, and 4% vinegar formulations (as % of total ingredient weight). Additional trials examined the effect of 1.7%, 2.2%, and 2.7% salt in the marinade formulation.

Trials included either 3 replicative trials with 2 samples taken at each testing period, or 2 replicate trials with 3 samples taken at each test period (*n* = 6/testing period) as per USDA-FSIS [28] and National Advisory Committee on the Microbiological Criteria for Foods (NACMCF) [25] guidelines.

#### 2.4.2. Extended Overnight Biltong Marination Process

Several extended marination procedures were also examined. One included a 1 h hold at 5 °C after the antimicrobial dip followed by 40 min vacuum-tumbling in a complete meat-spice-salt-vinegar marinade mixture (formulation: 95–96% beef, 2.5% spices includes salt at 2% overall formulation, and 2% 50-grain red wine vinegar), and then the marinaded beef was held for an extended period (overnight, ~16–18 h) at 5 °C (41 °F). This process examined if further reduction of *Salmonella* could be achieved by an extended refrigerated hold period after antimicrobial/marination treatment as was demonstrated previously with *E. coli* O157:H7 [29]. After the extended marination period, the beef pieces were removed from the excess vinegar marinade and hung in the humidity chamber at 23.9 °C (75 °F) and 55% RH and sampled after 0 (post-marinade), 2, 4, 6, and 8 days of drying.

Another extended marination procedure involved non-vacuum tumbling (5 min) with just spices and salt (formulation: 95–96% beef, 4–5% spice which included salt at 2.1% of total dry formulation). Spiced beef pieces (dry) were then placed in stainless steel pans, and liquid marinade was slowly poured in for the beef to sit in while held at refrigeration temperature (5 °C/41 °F) overnight. Liquid marinade comprised 14% of total formulated weight (including beef); 50-grain white vinegar comprised 73% of the liquid marinade portion of the formulation and 10% of the total formulated weight. Marinaded beef pieces were then ‘turned’ after 30-min and again after 8–12 h and left to marinade for the remaining time in the refrigerator (total, 16–20 h). After marination, beef pieces were removed from the excess vinegar marinade and hung in the humidity chamber (22.8 °C/73 °F, 55% RH) as described previously and sampled at 0 (post-marinade), 2, 4, 6, and 8-days of drying.

#### 2.4.3. Biltong Drying Process

After marination by either the short term or the longer extended processes, beef pieces were hung using large paper clips in a 10 cubic foot benchtop humidity oven (Hotpack Model #435315, SP Industries, Warminster, PA, USA) set at 22–25 °C (72–77 °F) depending on the process, and 55% RH. Relative humidity was established by a direct water line feed from an in-house deionized water supply to the built-in bottom water chamber with automatic water level and heating element to warm the water and generate humidity; an internal fan circulated the air within the chamber. Although air temperature and humidity level control was built-in, additional external monitors were used consisting of a handheld humidity monitor (Vaisala HM70, Helsinki, Finland) and a handheld thermocouple temperature recorder (Center 378, New Taipei City, Taiwan) with 4 temperature probes for chamber temperature (2 probes) and beef temperature (2 probes). A laptop computer was used to record temperatures and humidity during the entire process (Figure 1O–P).

### 2.5. Water Activity and Moisture-Loss Determination

**Figure 2:**
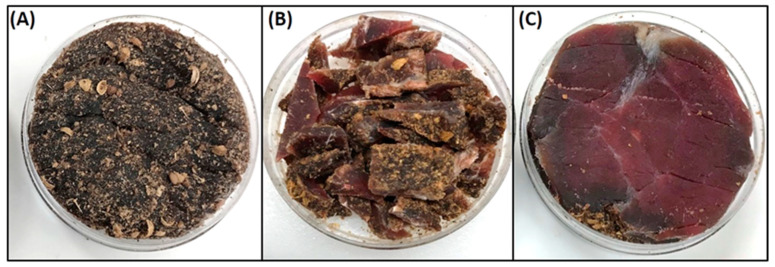
Water activity measurements. Examination of all possible samples from which water activity could be derived using the cupules for the water activity meter: (**A**) outer surface, (**B**) chopped pieces, and (**C**) inside surface. The sensor of the water activity meter would be positioned just above the retained sample in the cupule holder.

Water activity was measured using a HC2-AW-USB probe with direct PC interface and HW4-P-Quick software (Rotronic Corp., Hauppauge, NY, USA) (Figure 1Q). Samples for water activity and moisture loss were obtained using negative controls (i.e., non-inoculated beef, dipped, marinaded, and tumbled) during various stages of the process: the initial raw beef, beef after the spice/salt/vinegar marination step, and beef after 2, 4, 6, and 8–9 days of drying in the humidity chamber. Samples were cut in half and the interior portion of the biltong beef samples were positioned with the inside cut surface facing upward in the sampling cupules (Figure 1Q), towards the water activity meter sensor, as this was the region of highest water activity even with biltong at the end of drying (Figure 1R). This was determined by comparing water activity taken from the surface side facing the sensor, the inside facing the sensor, and chopped up pieces of entire biltong pieces in the sample cupule (Figure 2). Samples from the refrigerator or drying oven were allowed to equilibrate to room temperature in the covered cupules before analysis.

Moisture loss was determined by weighing beef pieces (negative controls) prior to the start of drying and again after drying for 2, 4, 6, and 8 days. Comparison of latter weights to initial weights of the same pieces resulted in determination of % moisture loss as per Equation (1):

(1) $$\%\;Moisture\;Loss=\frac{\left[\left(initial\;weight\right)-\left(latter\;weight\right)\right]\;}{\left(initial\;weight\right)}\times 100\;$$

### 2.6. Microbial Sampling and Enumeration of Beef

Beef samples were randomly chosen and transferred to 4-mil sterile Whirl-pak filter-stomaching bags (Nasco, Fort Atkinson, WI, USA), followed by addition of 100 mL of chilled 1% neutralizing buffered peptone water (nBPW, Criterion, Hardy Diagnostics, Santa Maria, CA, USA) and then stomached for 90 s in a masticator paddle-blender (IUL Instruments, Barcelona, Spain). The filter bag dilution (stomached sample) was considered the 10^0^ dilution for all samplings, including the initially inoculated raw beef through the final samples at up to 8–10 days of drying so that microbial counts were directly comparable with each other at all stages of drying. After stomaching in nBPW, inoculated (experimental) and non-inoculated (negative control) samples were 10-fold serially-diluted with 0.1% BPW. Dilutions were then surface plated (0.1 mL) in duplicate on TSA or selenite cystine agar (SCA). Both media contained spectinomycin (5 µg/mL), clindamycin (5 µg/mL), and novobiocin (50 µg/mL), and plates were incubated at 37 °C for 48 h before enumeration. In a prior study, SCA was shown to enumerate these same acid adapted *Salmonella* serovars comparably to TSA, even after exposure to different types of stress [21]. When microbial counts were expected to be low, 0.2 mL was plated on each of 5 plates (1 mL total) to increase the sensitivity of plating (i.e., decrease the limit of detection).

### 2.7. Statistical Analysis

Each trial was performed in either triplicate replication with 2 samples tested per sampling period (*n* = 6) or duplicate replications with 3 samples tested per sampling period (*n* = 6) in accordance with validation testing criteria established by the NACMCF [25] and accepted by USDA-FSIS [28]. All replications were performed as autonomous and separate experiments using separately inoculated cultures, separately prepared plating media, and meat from different animals. Data are presented as the mean of multiple replications with standard deviation of the mean represented by error bars. Statistical analysis of timed series data was undertaken using repeated measures one-way analysis of variance (RM-ANOVA) and the Holm–Sidak test for pairwise multiple comparisons to determine significant differences (*p* < 0.05) of the treatments. Data treatments with different letters are significantly different (*p* < 0.05); treatments with the same letter are not significantly different (*p* > 0.05).

## 3. Results

### 3.1. Short-Term Biltong Marination Process

**Figure 3:**
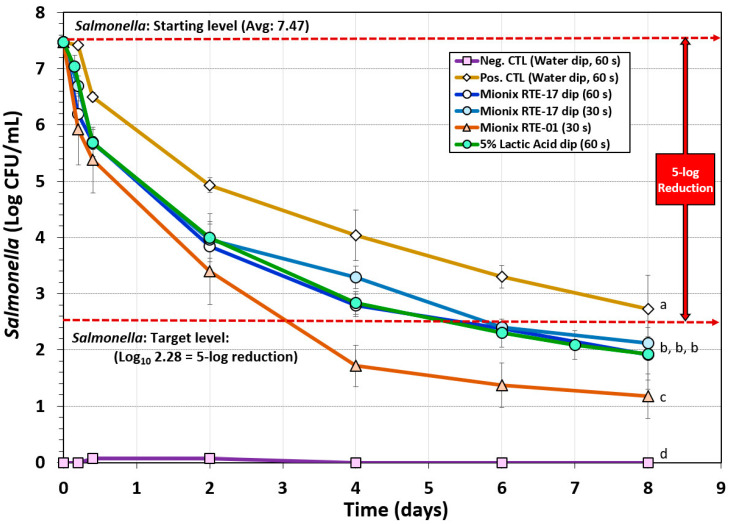
Short-term processing of biltong for reduction of *Salmonella*. Comparison of antimicrobials and pre-marination dip treatment time (30 or 60 s) with antimicrobials including lactic acid (5%) and acidified calcium sulfate (Mionix RTE-17 diluted to 5% lactic acid; Mionix RTE-01 diluted to 10% lactic acid). After treatment and marination, beef was held at 25 °C (77 °F) and 55% relative humidity (RH) for up to 8 days. Non-inoculated negative controls (Neg CTL) were used to demonstrate the effectiveness of selective media against background organisms. Graphs of different trials were adjusted to a common starting level. Treatments were analyzed by repeated measures one-way analysis of variance (RM-ANOVA) using the Holm–Sidak test for pairwise multiple comparisons to determine significant differences; treatments with different letters are significantly different (*p* < 0.05); treatments with the same letter are not significantly different (*p* > 0.05).

A biltong process using a short marination process (i.e., a 30 min vacuum tumbling) facilitated process completion the same day it was initiated including hanging beef pieces in the humidity chamber. The data show that dip treatment in antimicrobials such as acidified calcium sulfate adjusted to 5% lactic acid (Mionix RTE-17) or 5% lactic acid (ADM FCM88), prior to marination, resulted in a ~5.0-log reduction of *Salmonella* in 6 days and ~5.5-log reduction by 8 days (Figure 3). The trials with acidified calcium sulfate (at 5% lactic acid) and 5% lactic acid were nearly identical and showed no significant difference (*p* > 0.05). Acidified calcium sulfate (Mionix RTE-01) adjusted to 10% lactic acid showed a steeper decline in reduction of *Salmonella* while drying and reached >5-log reduction in 4 days and >6-log reduction by 8 days and was significantly different (*p* < 0.05) than 5% lactic acid (ADM FCM88) or acidified calcium sulfate (Mionix RTE-17) adjusted to 5% lactic acid (Figure 3). Although the inoculated positive control trials without additional antimicrobial dip treatment did not reach the targeted 5-log reduction in 8 days and was significantly different to trials with antimicrobial treatment (*p* < 0.05), it could likely have reached 5-log reduction level if given a few more days of drying (Figure 3); some individual replicates did reach this level within 8 days, but the average of all replications was slightly less than 5-log reduction.

**Figure 4:**
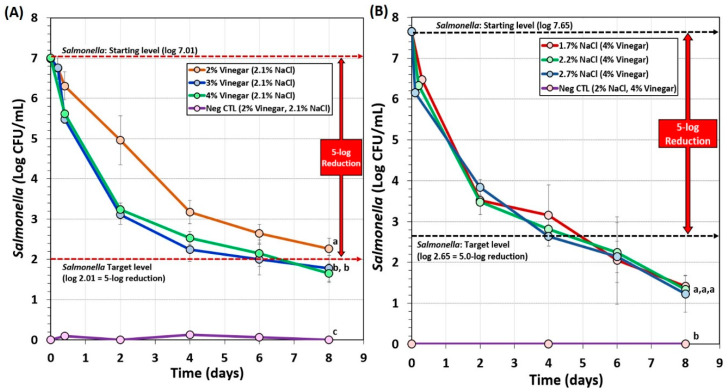
Short-term processing of biltong for reduction of *Salmonella*. (**A**) Comparison of 2%, 3%, or 4% vinegar in the marinade formulation. (**B**) Comparison of 1.7%, 2.2%, or 2.7% NaCl in the marinade formulation. After marination, beef was held at 25°C (77°F) and 55% RH for up to 8 days. Negative controls (Neg CTL) demonstrate the effectiveness of the selective media against background organisms from non-inoculated/processed beef. Graphs of various trials were adjusted to a common starting level. Treatments were analyzed by RM-ANOVA using the Holm–Sidak test for pairwise multiple comparisons to determine significant differences; treatments with different letters are significantly different (*p* < 0.05); treatments with the same letters are not significantly different (*p* > 0.05).

Biltong trials also examined the effect of vinegar level (2%, 3%, vs. 4% vinegar) and salt level (1.7%, 2.2%, or 2.7%) during marination on *Salmonella* lethality (Figure 4). The use of 2% vinegar in the marinade formulation again did not achieve 5-log reduction of *Salmonella* in 8 days of drying (Figure 4A). However, 3% and 4% vinegar demonstrated greater inhibition of *Salmonella*, dropping levels much earlier in the process yet showed no significant difference between them (*p* < 0.05) and both achieved >5-log reduction within 7–8 days (Figure 4A). Trials comparing the use of 1.7%, 2.2%, or 2.7% NaCl levels in the marinade formulation were not significantly different (*p* < 0.05) and all reached >5.5-log reduction in 6 days (Figure 4B). The data suggest that 1.7% NaCl may be used in order to reduce sodium levels in the final product.

### 3.2. Long-Term Biltong Marination Process

**Figure 5:**
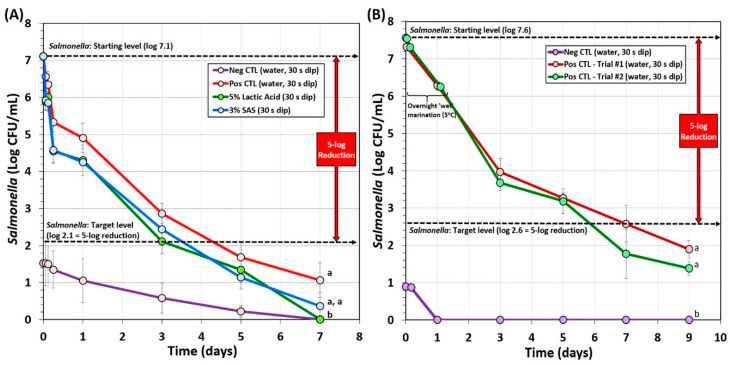
Processing of biltong for reduction of *Salmonella* involving overnight marination at 5 °C. (**A**) Overnight marination process including vacuum-tumbling of beef with spices, salt, and vinegar. Trials include pre-marination dip treatment with antimicrobials (5% lactic acid, 3% sodium acid sulfate) or water (positive and negative controls). After marination, beef was held at 23.9 °C (75 °F) and 55% RH for up to 6 days. (**B**) Overnight marination process, spiced-beef tumbled without vacuum and vinegar was added separately during static marination in the refrigerator. No separate antimicrobial dip treatment was used. After marination, beef was held at 22 °C (72 °F) and 55% RH for up to 8 days. Non-inoculated spice-processed beef (i.e., negative controls) was run in parallel with the other trials and plated on selective media. Graphs of different trials were adjusted to a common starting level. Treatments were analyzed by RM-ANOVA using the Holm–Sidak test for pairwise multiple comparisons to determine significant differences; treatments with different letters are significantly different (*p* < 0.05); treatments with the same letters are not significantly different (*p* > 0.05).

Several additional overnight marination processes were examined. One involved vacuum-tumbling with the complete marinade mixture followed by overnight marination at refrigeration temperature (Figure 5A). This process included microbial testing after inoculation, after an antimicrobial dip treatment and an additional refrigerated hold period (1 h), after an overnight refrigerated marination step (0 days drying), and after 2, 4, and 6 days of drying (Figure 5A). A 5-log reduction was achieved after 4 days of drying (Figure 5A). Background microorganisms that appeared on the ‘negative control’ plates (not inoculated with *Salmonella*) also declined during processing and were orders of magnitude lower than the levels of *Salmonella* on experimental samples inoculated with *Salmonella*. The data show that 5% lactic acid and 3% sodium acid sulfate both achieved 5-log reduction and were not significantly different (*p* < 0.05). Furthermore, positive control samples without additional antimicrobial dip treatment also achieved 5-log reduction, albeit later than the antimicrobial-treated samples (Figure 5A).

Another ‘overnight marination’ process included a traditional South African recipe whereby biltong beef samples were dry tumbled with spice and salt (without vacuum), placed in a pan, and vinegar was slowly added to prevent washing off the spices. This was marinaded overnight at 5 °C. The beef pieces sitting in the vinegar marinade (not completely covered) were turned over after 30–60 min, and then turned over again after 6–8 h until completion to allow equal marination of both sides prior to hanging in the humidity chamber. The extended overnight marinade provided a ~1.0–1.3 log reduction of *Salmonella* followed by an additional ~2.3-log reduction during the first 2-days of drying (Figure 5B). The targeted 5-log reduction of *Salmonella* was obtained after 6 days of drying (7th day on Figure 5B) and well beyond the 5-log reduction level by the 8th day. This method used the largest volume of vinegar (10% of total formulation) compared to the other methods.

### 3.3. Temperature and Relative Humidity Measurements

**Figure 6:**
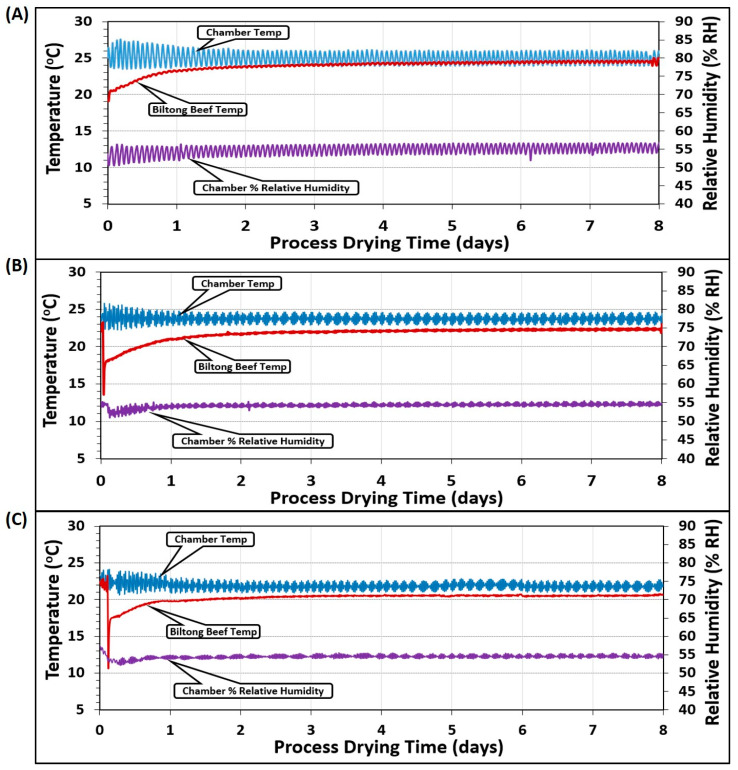
Temperature (°C) and relative humidity (% RH) during several different biltong processes covering three different ranges of temperature in the humidity oven: (**A**) 24–26 °C, (**B**) 23–24.5 °C, and (**C**) 21–23 °C, all maintained at 55% RH.

Temperature measurements were the average of 2 probes placed in the humidity oven chamber (chamber temp) and 2 additional probes inserted into 2 pieces of beef (beef temp) at different positions in the chamber (Figure 6). The various temperature/humidity parameters targeted 25 °C/55% RH (Figure 6A), 23.9 °C/55% RH (Figure 6B), and 22 °C/55% RH (Figure 6C). The chamber temperature varied more than the beef because the unit would heat up the air when the temperature fell below the set point and then refrigerate and cool down when it ran above the set point, showing an oscillation over time. The solid beef pieces were buffered from these short temperature changes and probes inserted into the beef did not show the same type of incremental variation. Similarly, an external humidity probe was inserted into the chamber that was set at the same 55% RH for each of the 3 different temperature regimens (Figure 6A–C).

### 3.4. Water Activity and Moisture-Loss Measurements

Water activity (A_w_) measurements were obtained using a Rotronic USB probe chamber (Figure 1Q), laptop, and software. Although many people suggested ‘chopping’ the biltong beef into pieces to get an overall ‘average’ of the product, it was determined that cutting the biltong beef so that the inside portion was tested for A_w_ provided the most conservative determination of water activity. Samples of the outer surface tested for water activity gave A_w_ ranges of 0.80–0.81 (Figure 2A). When biltong beef was positioned with the innermost portion directed upwards towards the sensor, A_w_ ranges of 0.89–0.90 were obtained (Figure 2C). Furthermore, when biltong beef was chopped up to have a combination of inner and the drier outside portions (Figure 2B), intermediate levels between these were observed (0.84–0.86).

**Figure 7:**
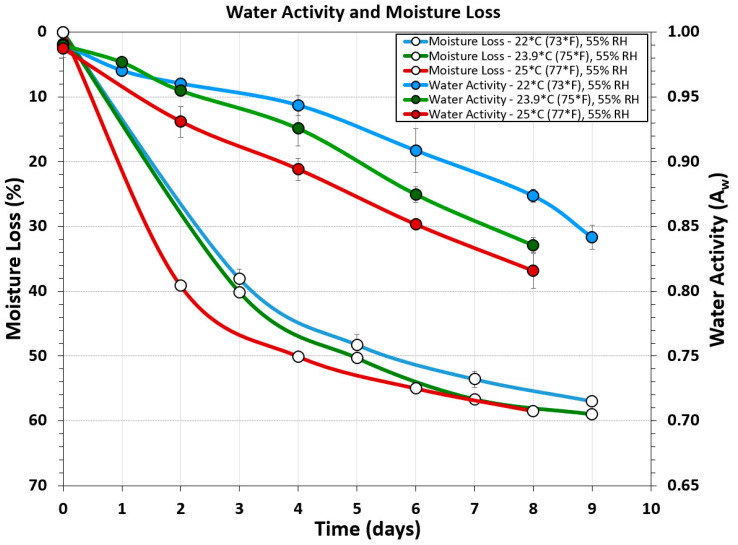
Water activity and moisture loss during several different biltong processes covering three different ranges of temperature at the same humidity level: 22 °C (73 °F), 23.9 °C (75 °F), and 25 °C (77 °F); all at 55% RH.

Water activity measurements were obtained using non-inoculated ‘negative control’ samples to alleviate concerns of *Salmonella*, but were still processed with the same spice, salt, and vinegar marinade as the positive controls. Water activity and moisture loss measurements taken from 3 different processing temperature ranges (22 °C, 23.9 °C, and 25 °C) but the same relative humidity (55% RH) show incremental decrease in both A_w_ and moisture loss over time as processing temperature is increased (Figure 7).

## 4. Discussion

Biltong is a new and expanding product line in the US dried beef market and is often marketed as a gourmet dried beef because it is not heated to high temperatures like beef jerky. Since it is an RTE shelf-stable dry beef, it must comply with USDA-FSIS guidelines (Revised Appendix A [30] and Jerky Guidelines [10]) that address *Salmonella* reduction during the manufacture of such products. The fact that ‘traditional’ South African biltong does not use a heat lethality step was viewed as making it difficult to achieve the targeted reduction of *Salmonella* with this product.

One of the main reasons a 5-log reduction of acid adapted *Salmonella* was so readily achieved in this study was the manner in which *Salmonella* was enumerated. In a prior study on biltong Burnham et al. (2008) enumerated *Salmonella* on a ‘colony forming unit per gram’ (cfu/g) basis from start to finish in their process, and they never achieved a 5-log reduction [16]. Jones et al. (2019) also enumerated on a cfu/g basis in their biltong study, but they did not evaluate log reduction of *Salmonella*, but simply analyzed for log presence of various microorganisms on biltong at the end of the process [5]. They were processing according to South African regulatory standards which does not require a specified log-reduction of targeted microorganisms. Similar enumeration on a cfu/g basis has also been reported for numerous studies with beef jerky [4,6,31,32]. The comparison of microbial enumeration (i.e., cfu/g) between fresh raw beef (100% moisture) at the beginning of the process to the dried product at the end of the process (~60% moisture loss) is an inequitable microbial comparison. Drying of the underlying beef results in a concentration of residual microbial counts that undercuts the log reduction of the remaining *Salmonella* because the microorganisms are concentrated if enumerated on a ‘per gram’ basis. In this study, each similar-sized piece of beef was inoculated with a fixed quantity of inoculum (i.e., 150 µL/side; 300 µL/piece). Enumeration was then obtained by stomaching fresh (~100–110 g), partially-processed (~90–70 g), or dried beef (~50–40 g) in the same fixed volume of recovery diluent (i.e,., 100 mL), thereby overcoming problems due to concentration of the underlying beef tissue (and the overlying microbial population) by moisture loss. By maintaining a fixed volume of diluent, the microbial concentration factor is overcome and the final count is directly related to the earlier count without influence of beef tissue concentration due to drying.

Although traditional biltong processing does not include a thermal lethality step, it generally includes several ingredients (salt, vinegar) and processing conditions (drying) that either individually, or in combination, provides an antimicrobial effect. Salt is one of those ingredients that is not restricted, although the US FDA and Health and Human Services (HHS) have put in place voluntary programs to reduce the use of sodium because of overuse and excess consumption in the US food supply has resulted in high levels of hypertension and chronic heart disease [33]. In dried beef products, externally applied salt in the marinade helps to bind moisture and draw water out of meat (and bacteria) to facilitate drying. The use of different levels of sodium chloride (1.7%, 2.2%, and 2.7%) showed no significant difference (*p* < 0.05) in *Salmonella* lethality during biltong processing and, therefore, the use of 1.7% NaCl would do well to maintain *Salmonella* lethality while simultaneously reducing sodium levels in the finished product.

In the USA, vinegar and acetic acid are not considered equivalent ingredients, even at the same level of acetic acid concentration. On meat products, the application of acetic acid is regulated according to the USDA-FSIS ‘Safe and Suitable Ingredients List’ as a processing aid [27]. The USDA-FSIS does not regulate the use of vinegar because it is covered by US FDA regulations [34] as a Generally Recognized as Safe (GRAS) substance with no restriction on concentration limit (as ‘grain’ or % acetic acid) or treatment time. Thereby marination with vinegar can be short or as long as an overnight process. However, a process validated at a lower concentration of vinegar can be readily switched to a higher vinegar concentration, but the reverse is not true unless microbial (*Salmonella*) validation data is provided to justify the decrease. In contrast, the use of ‘acetic acid’ as a processing aid may be limited to a particular concentration (i.e., ≤5%) and an ‘appropriate treatment time’ (i.e., 30–60 s is appropriate; 10 min is not appropriate). When vinegar is used, it must be listed on the ingredient label and the particular type of vinegar must be specified (i.e., white distilled vinegar, apple cider vinegar, balsamic vinegar, cane vinegar, coconut vinegar, malt vinegar, red wine vinegar, rice vinegar, sherry vinegar, white wine vinegar). Although vinegar is required to be listed on the ingredient label, it is generally considered an acceptable and innocuous ingredient by most consumers.

Additional antimicrobials may also be used to improve pathogen reduction prior to trimming or on the subsequent trimmed beef strips. Many of the preferred antimicrobials used on meat and poultry in the USA as per the USDA-FSIS Safe and Suitable list [27] are those that are considered ‘processing aids’ [35]. Certain antimicrobials that can be designated as processing aids have restricted use levels and contact times, and if used as designated by USDA-FSIS, they are not required to be listed on the ingredient label. According to federal labeling requirements, processing aids are defined as, “substances that are added to a food for their technical or functional effect in the processing but are present in the finished food at insignificant levels and do not have any technical or functional effect in that food” [36,37] and, therefore, are not required to be labeled. Of the antimicrobials used in this study, acidified calcium sulfate (RTE-01 at 10% lactic acid) would have to be listed on the label. However, lactic acid (5%), sodium acid sulfate (3%), and acidified calcium sulfate (RTE-17 at 5% lactic acid) are considered processing aids and need not be listed on the ingredient label in the USA. This type of ‘clean/green label’ ingredient is preferable to many companies that do not want excessive and complicated ingredient labels.

## 5. Conclusions

The absence of a heat lethality step during biltong processing and the inability of a prior biltong study to achieve 5-log reduction lead us to believe that a 5-log reduction of *Salmonella* in biltong would be difficult to obtain and initially we resorted to the use of additional antimicrobial treatments to achieve these reductions. The combination of vinegar/salt marinade by itself appears to give a significant reduction during processing and subsequent drying. Perhaps even more important was the manner of microbial enumeration that eliminated the effects of dried beef substrate that reduces the log reduction if performed on a cfu/g basis. We were able to demonstrate a 5-log reduction of *Salmonella* in all trials involving different processes and different ingredient formulations, even with the use of acid adapted cultures with reduced sensitivity to acidic conditions (i.e., vinegar) and without additional antimicrobials.

## Figures and Tables

**Table 1:** Antimicrobials used in this study as listed in the United States Department of Agriculture Food Safety and Inspection Service (USDA-FSIS) Safe and Suitable Ingredients List (7120.1).

Category	Substance	Intended Use of Product	Amount	Label Key ^a^
Antimicrobials Lactic acid, 5%	Lactic acid (5%).	Beef and pork sub-primals and trimmings.	2% to 5 % solution of lactic acid, not to exceed 55 °C.	None under the accepted conditions of use (1)
Antimicrobials Sodium acid sulfate, 3%	An aqueous solution of sulfuric acid and sodium sulfate.	In the form of a spray, wash, or dip on the surface of meat (beef and pork) and poultry products processing.	Solution of sulfuric acid and sodium sulfate at concentrations sufficient to achieve a targeted pH range of 1.0–2.2 on the surface of meat and poultry.	None under the accepted conditions of use (1)
Antimicrobials Acidic calcium sulfate (Mionix RTE-17, 5% lactic acid)	An aqueous solution of acidic calcium sulfate and lactic acid.	Applied as a continuous spray or a dip on raw poultry carcasses, parts, giblets, and ground poultry.	Acidic calcium sulfate sufficient for purpose; lactic acid not to exceed 5.0 % and 55 °C.	None under the accepted conditions of use (1)
Antimicrobials Acidic calcium sulfate (Mionix RTE-01, 10% lactic acid)	A solution of water, acidic calcium sulfate, lactic acid, and sodium phosphate (solution with a pH range of 1.45 to 1.55).	Raw whole muscle beef cuts and cooked roast beef and similar cooked beef products (e.g., corned beef, pastrami, etc.).	A solution of water, acidic calcium sulfate, lactic acid, and sodium phosphate (solution with a pH range of 1.45 to 1.55) spray applied for up to 30 s of continual application *sodium phosphate on finished product must not exceed 5000 ppm.	Listed by common name in ingredients statement of multi-ingredient products. Single ingredient raw whole muscle beef cuts must be descriptively labeled (2)

**^a^ Food ingredient label key:** (1) The use of the substance(s) is consistent with the Food and Drug Administration’s (FDA) labeling definition of a processing aid, (2) Generally Recognized as Safe (GRAS). The USDA-FSIS regulates which antimicrobials, dependent on use level and application time, must be included on food ingredient labels or can be excluded from such labels because their level and treatment time satisfies the definition of a ‘processing aid’.
